# Constitutive expression of Asparaginase in *Gossypium hirsutum* triggers insecticidal activity against *Bemisia tabaci*

**DOI:** 10.1038/s41598-020-65249-w

**Published:** 2020-06-02

**Authors:** Ambreen Gul, Ghulam Hussain, Adnan Iqbal, Abdul Qayyum Rao, Salah ud Din, Aneela Yasmeen, Naila Shahid, Ammara Ahad, Ayesha Latif, Saira Azam, Tahir Rehman Samiullah, Samina Hassan, Ahmad Ali Shahid, Tayyab Husnain

**Affiliations:** 1 Centre of Excellence in Molecular Biology, University of the Punjab, 87-West Canal Bank Road, Lahore, 53700 Pakistan; 2 Kinnaird College for Women University, Lahore, Pakistan; 3 Molecular Biology and Genetics, Aarhus University, Aarhus, Denmark

**Keywords:** Biotechnology, Molecular biology, Plant sciences

## Abstract

Whitefly infestation of cotton crop imparts enormous damage to cotton yield by severely affecting plant health, vigour and transmitting *C*otton *L*eaf *Cu*rl *V*irus (CLCuV). Genetic modification of cotton helps to overcome both the direct whitefly infestation as well as CLCuV based cotton yield losses. We have constitutively overexpressed asparaginase (*ZmASN*) gene in *Gossypium hirsutum* to overcome the cotton yield losses imparted by whitefly infestation. We achieved 2.54% transformation efficiency in CIM-482 by *Agrobacterium*-mediated shoot apex transformation method. The relative qRT-PCR revealed 40-fold higher transcripts of *asparaginase* in transgenic cotton line vs. non-transgenic cotton lines. Metabolic analysis showed higher contents of aspartic acid and glutamic acid in seeds and phloem sap of the transgenic cotton lines. Phenotypically, the transgenic cotton lines showed vigorous growth and height, greater number of bolls, and yield. Among six representative transgenic cotton lines, line 14 had higher photosynthetic rate, stomatal conductance, smooth fiber surface, increased fiber convolutions (SEM analysis) and 95% whitefly mortality as compared to non-transgenic cotton line. The gene integration analysis by fluorescence *in situ* hybridization showed single copy gene integration at chromosome number 1. Collectively, asparaginase gene demonstrated potential to control whitefly infestation, post-infestation damages and improve cotton plant health and yield: a pre-requisite for farmer’s community.

## Introduction

L-asparaginases play an important role in bacteria, fungi, plants, and the tissues of some animals excluding humans^1^. In plants, asparaginases are key players in regulating asparagine (primary N source) by releasing ammonia and aspartate via the deamidation pathway, which is the preferred route for developing plant organs. The aspartic acid resulting from this pathway is incorporated into the aspartate family of amino acids^2,3^. There are two reported categories of asparaginases: plant and microbial. Plant asparaginases are further subdivided into two subclasses based on their dependency on potassium (K^+^): K^+^-dependent and K^+^-independent. These are isolated from various plants including *Arabidopsis thaliana*, *Lupinus albus*, *Lupinus polyphyllus*, *Phaseolus vulgaris*^4^, *Lupinus arboreus*, *Lupinus angustifolius*^5^, *Lotus japonicus*^6^, *Glycine max*^7^, *Withania somnifera*^8,9^, and *Pisum sativum*^10^. K^+^-dependent asparaginases exhibit asparaginase activity^11,12^ whereas K^+^-independent asparaginases (isolated from *Lupinus luteus* (LlA)) possess both isoaspartyl peptidase and asparaginase activity and recognize β-aspartyl-His as a substrate^3,13^. Under nitrogen-limiting conditions, the stored asparagine is the main source of nitrogen for the growing parts of plants, i.e., developing leaves and seeds^14^.

A total of 81 % of the global cotton fiber stems from genetically modified cotton; GM cotton helps combat various biotic and abiotic stresses^15^ that negatively affect the quality and quantity of fiber^16^. In 2017, Rahman *et al*. (2017) reported that approximately 50–60% of the cotton fibers were lost due to the attack of a major feeding pest, silverleaf whitefly (*Bemisia tabaci)*^17^. An infestation of *Bemisia tabaci* results in modulations to the expression of various proteins involved in the TCA cycle, carbohydrate metabolism, photosynthesis, carbon fixation, and reactive oxygen species balance^18,19^. Pest infestation activates the salicylic acid signaling pathway followed by jasmonic acid signaling pathway^20,21^. Both these defense systems prepare the plant for upcoming challenges by modifying its carbon and nitrogen metabolism. Whitefly feeding elicits pathogenesis-related defense signals- it promotes salicylic acid signaling proteins instead of eliciting jasmonic acid-related proteins^18,19^. Pathogen-induced metabolic perturbations have shown increase in Val, Leu, Ile, Phe, Tyr, Trp and Lys while decrease in Asp^21^. Fall armyworm larvae, resistant to *Cry1F* toxic protein, accumulate more Asn in their midgut^22^. Interestingly, pathogen resistant finger limes had higher contents of Asn, Ile, Ser, Phe, and 2-aminobenzoic acid, thus predicting their role in the plant defense against pathogens^23^.

Previous researches were addressed to improve the nutritional deficiencies of plant by manipulating aspartic acid synthesis pathway^24,25^ and in decreasing the asparagine concentration, which is acrylamide precursor– a cancer-causing agent^14^. To the best of our knowledge, no one has considered the role of aspartic acid/glutamic acid against the feeding-insect related plant defense systems. In the present study, we have focused on the overexpression of aspartic acid by CaMV35S regulated asparaginase gene from *Zea mays (ZmASN)*. The gene construct was transformed into cotton using *Agrobacterium*-mediated transformation. The pathway of aspartate, glutamate, glutamine, and asparagine were analyzed and their simultaneous effect on the plant’s health and insect resistance were evaluated.

## Results

### Secondary structure analysis of *ZmASN* protein

**Figure 1:**
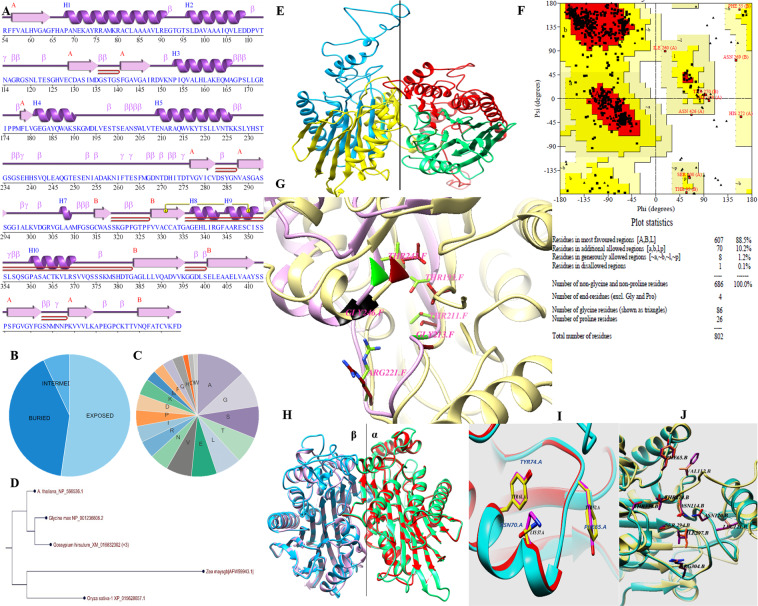
Bioinformatics analysis of *ZmASN* Protein. (**A**) Secondary structure containing both alpha and beta subunits. Protein motifs include: 2 sheets (A and B), 1 βαβ unit, 6 β hairpins , 3 β bulges, 13 β strands , 10 helices , 10 helix-helix interacts, 46 β turns, 9 γ turns, and 1 disulphide bond . (**B**) Solvent accessibility profile depicting 52.3% exposed, 40.46% buried, and 77.07% intermediate amino acid residues. (**C**) Amino acid composition shows 13.07% alanine, 9.54% glycine, 8.48% serine, 7.07% threonine, 6.71% lysine, 6.71% glutamic acid, 6.71% valine, 4.95% asparagine, and the remainder others. (**D**) Phylogenetic analysis of *ZmASN* protein with *Oryza sativa*, *Arabidopsis thaliana*, *Gossypium hirsutum*, and *Glycine max*. (**E**) Cartoon representation of subunit α shown in blue (chain A) and red (chain C) and subunit β in yellow (chain B) and green (chain D). (**F**) Ramachandran plot analysis of *ZmASN* protein model. The plots statistics is shown at the bottom of the plot. (**G**) Super positioning of active sites of *ZmASN* (yellow ribbon) and 2gez.1 (pink ribbon). Conserved residues of the active site of both proteins are labelled (2gez numbering). (**H**) Stereodiagram of the superposition of *ZmASN* and 2a8i.1. Superposition of subunits α and of subunits β of both proteins; subunit α of 2a8i3 is shown in red and subunit β is shown in purple. The *ZmASN* subunit α is shown in spring green and subunits β in blue; the RMSD value is 0.14 Å. (**I**) Subunit α comparison with a color coding of **2a8i**) red and magenta; ***ZmASN*:** cyan and yellow. (**J**) Subunit β comparison with a color coding of 2a8i: cyan and purple; ***ZmASN*:** yellow and coral.

The *ZmASN* protein (283aa) with accession number gb|AFW59943.1 was retrieved in 2013 and is now available at NCBI site with accession number gb|AQK47335.1 (460aa). The older version encoded one of the two subunits of the dimeric precursor protein while the newer version encodes both subunits. The molecular weight of the protein monomer was found to be 29.83 kDa with a pI of 5.538 and cytoplasmic subcellular location by PredictProtein analysis tool. The *ZmASN* protein monomer consisted of 2 sheets, 1 βαβ unit, 6 βhairpins, 3 β bulges, 13 strands, 10 α-helices, 10 helix-helix interacts, 46 β turns, 9 γ turns, and 1 disulfide bond (Fig. 1A). The solvent accessibility profile revealed 52.3% exposed, 40.46% buried, and 7.07% intermediate amino acid residues (Fig. 1B). The amino acid composition analysis revealed that it is composed of 13.07% alanine, 9.54% glycine, 8.48% serine, 7.07% threonine, 6.71% lysine, 6.71% glutamic acid, 6.71% valine, 4.95% asparagine, and the rest all other amino acids (Fig. 1C). The *ZmASN* phylogenetic analysis confirmed the asparaginase-2 superfamily of plant asparaginases with a sequence identity of 35, 34, 34, and 30% with *Oryza sativa*, *Arabidopsis thaliana*, *Gossypium hirsutum*, and *Glycine max*, respectively (Fig. 1D).

### Homology modelling and comparative analysis of *ZmASN* protein and active site

The quaternary structure of the *ZmASN* protein shows its tetrameric nature with two α and two β subunits (Fig. 1E). The quaternary model of *ZmASN* was evaluated with Ramachandran plot. The Ramachandran plot analysis of the assembled *ZmASN* protein model shows that 88.5% residues fall in the favored region while 0.1% residues in the disallowed region are far from the active site and substrate binding residues of the protein. PROCHECK analysis indicated that the phi and psi angles of 10.2% of the residues fall in the additional allowed region and 1.2% residues were in generously allowed regions (Fig. 1F). The active site analysis of the *ZmASN* protein when compared with that of PDB ID: 2a8i.1 and 2gez.1(potassium independent asparaginase) reveals that *ZmASN* is comparable to that of 2gez.1 and the active site residues are conserved between the two but not with that of 2a8i (Fig. 1G), indicating the presence of asparaginase activity in *ZmASN* protein. The superposition of *ZmASN* with 2a8i (human taspase1) shows that the protein is conserved as hetero tetrameric protein which has an RMSD value of 0.14 Å depicting the overall reliability of the *ZmASN* protein model (Fig. 1H). Super-positioning of the *ZmASN* subunits α (Fig. 1I) and β (Fig. 1J) with that of 2a8i reveals all conserved residues). This analysis clearly indicated that *ZmASN* is a functionally stable protein and belongs to potassium independent family of plant asparaginases.

### Development of pCAMBIA35S*-ZmASN* transgenic lines

Cloning of the gene *ZmASN* into pCAMBIA 1301 vector was confirmed by restriction digestion with *Sac*I and *Hind*III restriction enzymes with a digested fragment size of 1562 bp (Supplementary IA**)**. The recombinant plant expression vector pCAMBIA35S_*ZmASN* was transformed into *Agrobacterium tumefaciens* LBA4404 strain and the transformants were screened through colony PCR with gene specific primers (Supplementary IB). The *Agrobacterium*-mediated transformation of CIM 482 cotton variety via shoot apex explant yielded 60 cotton plantlets with 2.54% transformation efficiency (Table 1). Ten out of fourteen putative transgenic plants in soil pots were confirmed positive through PCR with gene specific primers (Supplementary II). Eight of the transgenic cotton plants were studied through rest of the analysis in advanced generations.

**Table 1:** Transformation efficiency of cotton.

Seedlings in selection media	Plants survived in soil	Survival efficiency %	PCR positive plants in soil	Transformation Efficiency %
552	60	10.86	14	2.54

### Gene expression and metabolic analysis

**Figure 2:**
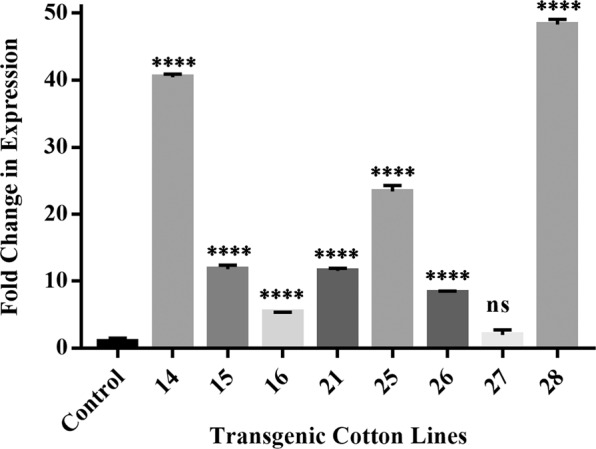
Relative expression of *ZmASN* gene in the young leaves of different transgenic lines. Mean fold change was measured using GhAct as internal control. The bars represent the mean of three biological replicates, and three technical replicates for each biological replicate. Graph is plotted as mean + SD. The significance of the data is determined by one-way ANOVA and Dunnett’s multiple comparison test at p < 0.0001 and is indicated by **** above bars whereas ns represents non-significant.

**Figure 3:**
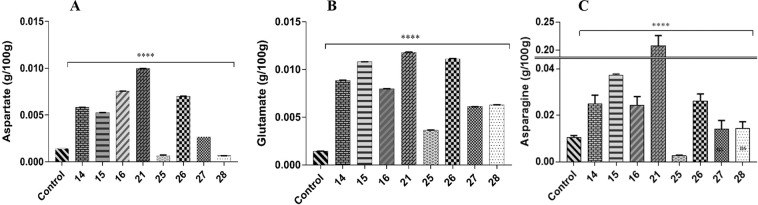
Quantification of free amino acid from cotton seeds of transgenic cotton lines and non-transgenic cotton lines in the T0 generation. (**A**) Free or unbound aspartic acid concentration. (**B**) Free or unbound glutamic acid concentration. (**C**) Free or unbound asparagine concentration. The bars represent the mean of five biological replicates and two technical replicates of each biological replicates. Graphs are plotted as mean + SD. The significance is determined by two-way ANOVA and Dunnett’s multiple comparison test at p < 0.0001 and is indicated by ****.

**Figure 4:**
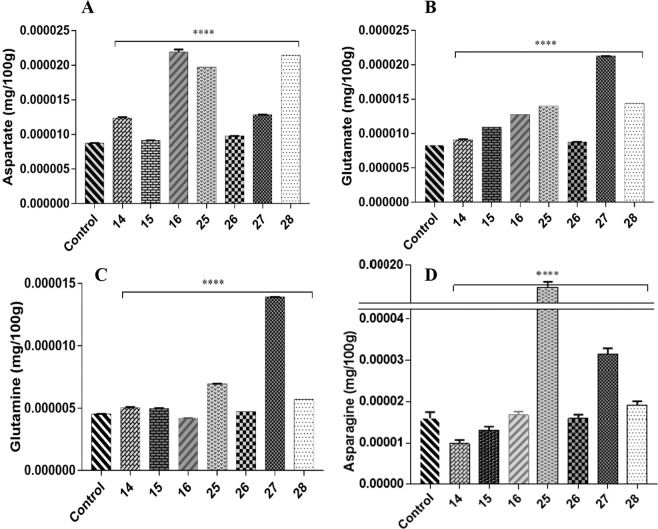
Quantification of free amino acid from phloem sap of transgenic cotton lines and non-transgenic cotton lines in the T1 generation. (**A**) Free or unbound aspartic acid concentration. (**B**) Free or unbound glutamic acid concentration. (**C**) Free or unbound asparagine concentration. (**D**) Free or unbound glutamine concentration. The bars represent mean of five biological replicates and two technical replicates of each biological replicates. Graphs are plotted as mean + SD. The significance is determined by two-way ANOVA and Dunnett’s multiple comparison test at p < 0.0001 and is indicated by ****.

Gene expression analysis with qRT-PCR using actin (GhAct4)^26^ as housekeeping gene revealed an increase in relative mRNA concentration in all transgenic cotton lines compared to non-transgenic control using method by^27^. The line 14 and 28 had 40- and 48-folds higher mRNA expression respectively while lowest relative mRNA expression was observed in transgenic cotton line 27 (Fig. 2). Because aspartic acid is readily converted to both glutamic acid and asparagine therefore, estimation of aspartate, glutamate, asparagine and glutamine concentration through UPLC (Ultra Performance Liquid Chromatography) from the seeds (T0) and phloem sap (T1) was performed. It showed an overall increase of aspartic acid in all transgenic cotton lines in comparison to non-transgenic cotton lines. Due to the constitutive expression of the gene, the seeds (T0 generation) of transgenic cotton lines, showed an increase in free aspartic acid/glutamic acid in lines 14, 15, 16, 26, 27 and 28 than the non-transgenic control (Fig. 3A,B). Cotton lines 25 and 28 were found to have boll worm infestation, while mild symptoms were observed on line 14, 15 and 27 especially at boll formation stage. Although the symptoms controlled through chemical pesticide, but it reflects low aspartate and glutamate in T0 generation of these plants. Cotton line 21 showed 8-fold increase of aspartate and total amino acid in the seed fraction **(**Fig. 3A**)**, however, we failed to raise its plants to maturity in T1 generation for this transgenic event. The free asparagine concentration remained comparable while free glutamine remained undetected in the seeds (T0) of transgenic cotton lines (Fig. 3C). In the phloem sap fraction (T1 generation), the cotton line 16 showed highest aspartate concentration, glutamate was also increased while asparagine and glutamine showed variable expression to that of non-transgenic cotton line. On an average, most of transgenic cotton lines were found to have higher aspartic acid (Fig. 4A) and glutamic acid (Fig. 4B) concentration than non-transgenic control. However, the asparagine concentration was found to be significantly reduced in cotton line 14 (Fig. 4D) while glutamine concentration (Fig. 4C) was higher than the non-transgenic control.

### Phenotypic analysis of transgenic cotton lines

**Figure 5:**
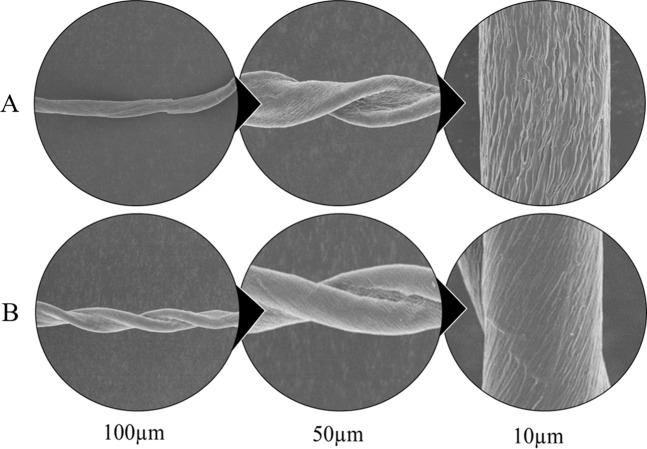
Scanning electron microscope images of mature cotton fiber grown under field conditions. (**A**) Mature cotton fiber of non-transgenic cotton plant scanned at 100 µm, 50 µm, and at 10 µm showing the fiber surface. (**B**) Mature cotton fiber of *ZmASN* transgenic plant scanned at 100 µm, 50 µm, and at 10 µm resolution showing the fiber surface.

Though the elevated aspartic acid concentration was the primary aim to control sucking insects; the impact of transgene insertion was also evaluated on the improvement of plant physiology and ultimately fiber yield of experimental plants. Phenotypically, transgenic cotton lines showed substantially increased plant height, number of bolls per plant, and fiber yield as compared to non-transgenic cotton plants. The fiber yield was higher in transgenic lines 14, 15, 25, and 26 in the T1 generation (Supplementary III). Leaf area of the transgenic cotton plants was comparable with non-transgenic cotton plants (Supplementary IV**)** indicating no relation to the transgene expression. Among the various fiber quality parameters, significant improvements in the micronaire values of line 14, 15, 25, and 27 were observed while the fiber length (UHML), uniformity index, and fiber strength remained comparable to that of control cotton lines (Supplementary V). It was observed that the *ZmASN* influenced the plant height, no. of sympodial branches, and yield of fiber. The effect of this gene was assessed on the surface chemistry of fiber. The SEM analysis of fiber surface of the transgenic cotton line 14 revealed smoother surface and increased convolutions than the control cotton plants (Fig. 5). The IRGA measurement of the physiological parameters i.e. photosynthetic rate (*A*), stomatal conductance (*g*_*s*_), and rate of transpiration (*E*) in the cotton line 14 were substantially elevated as 17.33 µmol m^−2^s^−1^,322 mmol m^−2^s^−1^, and 3.83 mmol m^−2^s^−1^ respectively as compared to the non-transgenic cotton line as 9.5 µmol m^−2^s^−1^, 37.66 mmol m^−2^s^−1^, and 0.4 mmol m^−2^s^−1^ respectively suggesting the role of this gene on physiological performance (Supplementary VI).

### *In planta* whitefly bioassay of *ZmASN* transgenic cotton plants

**Figure 6:**
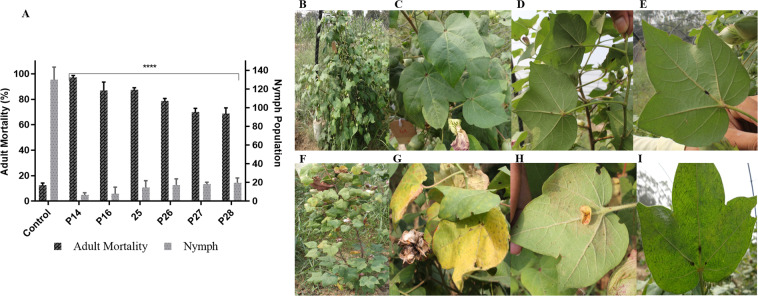
Whitefly bioassay of *ZmASN* transgenic lines (T2-generation). (**A**) Graph represents the % mortality of whitefly adults after 72hrs of infestation on non-transgenic control plants compared with different transgenic cotton lines and reduction in the number of nymphs on the control plant is compared with different transgenic cotton lines. Two-way ANOVA for statistical analysis at p < 0.0001 and Dunnett’s multiple comparison test was used to measure the level of significance as ****. The data was collected as n = 6 replicates. (**B**) Transgenic cotton plant after whitefly infestation. (**C**) Healthy leaves of transgenic cotton plant. (**D**) Bollworm infestation in transgenic cotton line and (**E**) No-virus after whitefly infestation. (**F**) Non-transgenic control cotton plant, **G**) Bollworm infestation on control cotton plant. (**H,I**) Stunted growth of plant and CLCuV symptoms after whitefly infestation on non-transgenic control cotton plant.

**Figure 7:**
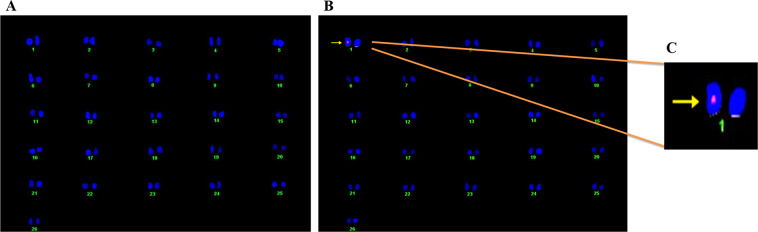
Fluorescence *In Situ* Hybridization (FISH) assay of *ZmASN* transgenic cotton plant. (**A**) Karyogram of non-transgenic control cotton plant performed on metaphase chromosomes without fluorescent signal. (**B**) Karyogram of *ZmASN* transgenic cotton plant showing the fluorescent signal determines the gene integration and location on the chromosome number 1. The single fluorescent signal is a depiction of hemizygosity of the integrated transgene. (**C**) Subset showing the transgene integration at chromosome 01.

The *in planta* clip-cage mortality assay was conducted on six representative lines (14, 16, 25, 26, 27, and 28) in T2 generation (2-month-old plants). The plants were infested with *Bemisia tabaci* Q-biotype^28^ (Supplementary VII) reared on CLCuV infected cotton plants. The 0–24 h old whitefly adults were released onto the representative transgenic cotton lines as well as non-transgenic control lines. Mortality data (F0) was recorded, and the development of nymphs of the next generation (F1) were observed on tag leaves. Transgenic cotton line 14 outperformed all other transgenic lines with highest (95.5%) adult whitefly mortality after 72 h, as compared to 10% mortality in control cotton line. The microscopic analysis of the tag leaves of assayed cotton lines (quarantined for additional 30 days) provided information about the nymph development in transgenic cotton lines. A perfect linear correlation of the adult mortality and nymph population was also recorded (+0.96). The nymph population in transgenic cotton line 14 reduced to 6 (minimum) while in transgenic cotton line 26 it reduced to 14 (highest). The nymph population on the non-transgenic control group was so enormous to be countable ~200/in^2^ (Fig. 6A). The field performance of the assayed plants was later observed under field conditions which showed the increase in height, and fiber yield (data not shown) as compared to non-transgenic cotton plants. After whitefly infestation, the transgenic cotton line 14 stood healthy (Fig. 6B) with early flowering, undetectable whitefly and CLCuV symptoms (Fig. 6C,E) and green leaves. However, the non-transgenic control cotton plants showed stunted growth (Fig. 6F), yellow leaves (Fig. 6G), whitefly infestation, developed the signs of CLCuV (Fig. 6H,I) and developed least number of bolls—less than 5 bolls per plant (data not shown). Figure 7 shows the karyogram of chromosomes in the metaphase stage where a single copy transgene in hemizygous form with single fluorescent spot on one chromatid of chromosome number 01 was observed in the transgenic cotton line 14 through FISH analysis with no signal on non-transgenic control plant.

## Discussion

Successfully produced transgenic crop plants should have minimum to no effect on crop biology and on nontarget insects, humans, and animals. The previous reports confirmed the neurotransmitter status of glutamate^29^, and its neurotoxic effects on the insect neuromuscular junctions^30–32^. However, the neurotransmitter and the neurotoxic status of aspartate (structural analog of glutamate) needs to be elucidated^32,33^. On the basis of preliminary *in vitro* feed based bioassays, in the current study, we investigated the toxic effects of aspartic acid on whitefly. The *ZmASN* protein was selected to increase the aspartate level in the plant sap so that its ability to induce toxicity in the whitefly after ingestion could be evaluated. The *ZmASN* protein model shows the αββα sandwich-like fold of asparaginase-2^2^ and the phylogenetic analysis also revealed the asparaginase family protein. Amino acid composition analysis shows that alanine, valine and leucine are major amino acids contributing to the hydrophobicity and stability of protein by van der Waals interactions. Glycine being the second highest amino acid provides flexibility and functional stability to the enzyme active site^34^. The crystal structure of *ZmASN* protein have not yet determined experimentally and for that reason the protein models were built by homology modelling and threading protocol. Before genetic transformation, these analyses were performed to ensure the proper conformation and folding of targeted protein. From homology modelling we can infers that the *ZmASN* protein has close resemblance to the human threonine aspartase (2a8i) while the presence of conserved residues of PDB ID:2gez (potassium-independent plant asparaginase) in the active site of the *ZmASN* protein confirmed that it is the K^+^-independent asparaginase with *Thr267* as its nucleophile, which is in accordance with the study by^2,35^ where Thr293 was the nucleophile. The *ZmASN* possess K^+^-independent L-asparaginase activity as determined by *in silico* analysis whereas due to its structural similarity to 2a8i (human taspase1) it may also possess isoaspartyl aminopeptidase activity. The recalcitrant nature of cotton is one of the biggest challenge for genetic transformation, despite the fact that 81% of worldwide cotton production is obtained from transgenic cotton^36^. In the current study, a transformation efficiency of 2.54% was achieved via the shoot apex cut method of transformation. The genetic makeup of the cotton explant’s wounding treatment, *Agrobacterium* strain and cell density, length of the transgene, and finally, the selection pressure of the antibiotic^37^ all contribute to transformation efficiency. The results are consistent with other studies where the transformation efficiency was recorded as, 1.25 %^38^. Higher transformation efficiency reported in other studies as (9%)^39^ and (20%)^40^ were only due to difference in efficiency calculation formula from current study. Native/endogenous expression of the asparaginase gene is the highest in young developing leaves and seeds^41^. In current study, the constitutive expression of *ZmASN* resulted in an increase of many-fold higher transcripts levels in mature plants which resulted in increase of both free aspartate and glutamate contents in both the seed and phloem sap of T0 and T1 generation plants respectively (Figs. 3A,B and 4A,B**)**. However, both aspartate and glutamate were found to be decreased in some of the transgenic cotton lines such as 25 and 28 which was assumed to be in response to severe pink bollworm infestation which causes stress on the host plant and modulate the expression of primary nitrogen metabolism enzymes^42^. Similar was the case with cotton lines 14, 15 and 27 especially at boll formation stage with mild symptoms (Fig. 6D), which was although controlled through chemical pesticide but still might impact concentration of both amino acid in these lines. These results are in accordance with the study by Curtin *et al*., (2018)^43^ who determined that free amino acid accumulation in the seed does vary considerably owing to the plant’s response to different stresses. The elevation in the *ZmASN* activity may not reduce the asparagine concentration (asparagine synthetase activity is not inhibited) while at the same time, glutamic acid and glutamine concentrations are directly affected by this *ZmASN* activity. However, as seed is primarily a storage compartment, and along with a change in aspartate concentration, the other three transport amino acids i.e. glutamate, glutamine and asparagine are differently affected. Moreover, in the seed fraction, free glutamine concentration was undetectable. Asparaginase hydrolyses asparagine into aspartic acid and an ammonia molecule, which is recycled by the GS/GOGAT cycle into glutamate or by aspartate aminotransferase into the glutamate, further elevating the glutamate content^41^. Consistent with this study, a similar increase in the concentration of aspartic acid in seeds via the overexpression of the AAT gene was reported^24,25^. Because every transgenic line is an independent event and the position of gene insertion in a tetraploid genome affects the translation and thus end-product accumulation differently, therefore, the aspartic acid concentration was weakly correlated (+0.43) with the gene expression data. Amino acids play an important role in the overall growth and physiology of the plant. Owing to the sensitivity of cotton plants towards environmental temperature, <25 ^o^C lead to substantial delays in fruit setting and cause yield reduction^16^. A considerable reduction in both the growth and fiber yield was observed during the study in both the control group as well as in all *ZmASN* transgenic cotton lines when the experiment performed at Denmark—an obvious phenomenon due to lower environmental temperature. Interestingly, under the same conditions, all transgenic cotton lines showed faster growth, increased height, number of bolls, and lint yield as compared to control group (which showed stunted growth). This clearly demonstrated the behavior of transgene on the growth of cotton plants while contradicting the results of^24,25^. An intricate balance of N supply and demand is a pre-requisite for healthy plant growth^41^. The increased asparagine concentration in a double mutant study resulted in root growth inhibition upon exogenous supply^44^. Similarly, asparagine and glutamine supplementation (0–1 mM) positively affected *Phaseolus vulgaris* growth, while >1mM concentrations negatively affected growth^45^. Fiber (cellulosic in nature) development is at the interplay of C and N metabolism, which are very closely interrelated^46^. The *ZmASN* transgenic cotton lines had 14.6% improved fiber strength while its micronaire value increased by 18.2% in the transgenic cotton line 14. Consistent with our study, aspartate, glutamate, and alanine concentrations were increased in both the transcriptome and metabolome of *G*. *hirsutum* at the fiber development stage^47^. Higher aspartate levels were also observed in the elongation stage of the fiber in the Ligon lintless-2 (*Li*_*2*_) mutant of cotton^48^. The effect of aspartate/glutamate on the development of fiber cell walls was observed in the form of smoother surface and an increase in the convolutions of fiber in the *ZmASN* transgenic cotton lines during the SEM analysis (Fig. 5), which was consistent with the studies by^48–50^. In *Vicia faba*, the enhanced stomatal opening was observed with an increase in the concentration of aspartate or glutamate^51^. Inter-dependence of carbohydrate synthesis pathways on the nitrogen assimilation pathway (which is modulated by our transgene) is supported by an increase in the photosynthetic parameters in *ZmASN* transgenic cotton lines.

The maximum mortality (95%) of whiteflies was observed in transgenic cotton line 14 in the clip-cage mortality assay. The *ZmASN* transgenic cotton plants remained healthy and resulted in maximum whitefly mortality upon whitefly infestation in comparison to the control non-transgenic cotton plants (Fig. 6). The results of this study are consistent with those of^52^, where the improvement in the nutritional status resulted in whitefly resistance in plants. High levels of glutamate cause insect neurotoxicity at neuromuscular junctions^31,32^ but the aspartate neurotoxicity was observed in our study whose regulation is probably governed by the same transporter. *In planta* whitefly mortality assay clearly demonstrated that higher concentration of aspartic/glutamic acid in transgenic cotton plants have significantly reduced adult whitefly population and minimum establishment of nymphs of next generation. The phenotypic analysis of the transgenic cotton plants sheds light on the interaction of aspartic acid on plant’s health and resistance to whitefly. While few transgenic lines (Line 28, line 16, line 26) showed very mild symptoms of CLCuV, but overall, the plants stayed healthy and all the phenotypic data of transgenic cotton plants showed faster plant growth and increase in fiber yield while on the other hand resistance to whitefly infestation and colonization was observed in the current study. This could be explained by the fact that extracellular levels of glutamate are regulated by excitatory amino acid transporters (EAAT). During sodium-coupled transport, aspartate is preferentially taken by this transporter, triggering higher accumulation in insect neuromuscular junctions^53^, congruous with the current study. This study reflects an excellent approach used for control of sucking insects like whitefly, which are a source of CLCuV infestation; this infestation results in a loss of 2 million bales each year in Pakistan. The transgenic material developed in the form of cotton seeds can be utilized in breeding programmes for variety development and improvement of the farmer community in countries such as Pakistan.

## Materials and methods

### Structural and functional analysis of the *ZmASN*

The *ZmASN* cDNA sequence, (gb|AFW59943.1) after being retrieved from NCBI database, owing to unavailability of cotton asparaginase in year 2013 during design of this study, was codon-optimized using the Integrated DNA Technologies (*IDT*) online tool. PredictProtein service was used for primary structure analysis^54^, ExPasy tool (https://web.expasy.org/compute_pi/) for protein molecular weight determination, and PDBsum tool for secondary structure prediction^55^. For automated protein modelling with SWISS-MODEL^56^, the template PDB ID: 2a8i showing 52.90% sequence identity was selected. The stereochemical properties of *ZmASN* individual subunits and the assembled model were predicted by a series of tests to evaluate its consistency and internal reliability. For the evaluation of a 3D predicted model, we used PROCHECK v3.4.4 for energy minimization, simulation, and refinement^57^. The dihedral Psi/Phi/Chi/Omega angles were assessed using the Ramachandran plot to see whether the values agree with the allowed conformation of the protein backbone. The model was superimposed on the crystal structures of PDB code: 2a8i.1 and 2gez.1 for the analysis of binding sites and active site residues of the *ZmASN* using CHIMERA v2013^58^.

### Plasmid construct

The gene cassette comprising 35S promoter, ADH enhancer, the *ZmASN* gene, and a Nos terminator in a 5′ to 3′ direction was synthesized from BioBasic Inc. (https://www.biobasic.com/us/gene-splash-gene-in-vector/). The *ZmASN* cassette was cloned at restriction sites *Sac*I/*Hind*III in plant expression vector pCAMBIA-1301, followed by screening of the transformed *E*. *coli*-Top10 strain with kanamycin (50 µg/ml) and tetracycline (50 µg/ml) selection on LB agar plates and then digestion of the plasmid with *Sac*I*/Hind*III restriction enzymes. The recombinant plasmid *pCAMBIA-ZmASN* was transformed into the *Agrobacterium tumefaciens* LBA4404 strain.

### *Agrobacterium-*mediated transformation of cotton shoot apices

Seeds of *Gossypium hirsutum* variety CIM-482 were obtained from the repository of the CEMB, University of the Punjab. Shoot apex explants were prepared by the method of^59^. The explants were preincubated in overnight grown *Agrobacterium* (OD_600_ = 1.0; cells harvested at 3000 × g, 4°C for 10 min; resuspended in basal MS-0 broth) at 28°C for 60 min in the dark with gentle shaking. The seedlings were then blot dried on sterile filter paper and transferred to MS-0 + cefotaxime (100 µg/ml) plates for co-cultivation. After 3–4 days, healthy seedlings were transferred to MS-tubes containing hygromycin (30 µg/ml), cefotaxime (100 µg/ml), B5-vitamins (nicotinic acid: 50 mM; thiamine-HCl: 10 mM; pyridoxine-HCl: 10 mM; myo-inositol: 100 mM; glycine: 2 mM) and incubated at 28 °C, 16 h light: 8 h dark, and at 60 μE m^−2^s^−1^ light for *in vitro* growth.

### Screening and acclimatization of putative transgenic cotton plants

The young leaves of putative transgenic cotton plants were used for genomic DNA isolation for PCR-based screening and the positive plants were shifted to plastic pots containing sterilized soil and covered with polythene bags to prevent dehydration. After gradual acclimatization to the open environment by removing the plastic bags at short intervals starting from 30 min up to one day the plants were hardened off in sunlight in a similar manner. The cotton flowers were self-crossed, and mature bolls were harvested.

### Molecular analysis of transgenic cotton plants

The genomic DNA purification was performed by the method of^60^ followed by PCR amplification with gene-specific primers. A modified CTAB-RNA extraction method by^61^ was used for total RNA isolation from the transgenic plants and cDNA was prepared using a Superscript II First Strand cDNA Synthesis kit (Thermo Fisher Scientific, cat#18064014) as per the manufacturer’s instructions. The mRNA expression of the transgene was analyzed by qRT-PCR using Maxima SYBR Green/ROX (cat # K0229). The 10 µl reaction mix containing 1 µl of both forward and reverse primers (5 µM), 6 µl of Maxima SYBR Green/ROX qPCR Master Mix (2×), 1 µl of cDNA template, and 3 µl of nuclease free H_2_O was prepared; GhAct4 was used data normalization. All primers for PCR, qRT-PCR, and GhAct4 are listed in Supplementary VIII. The real-time PCR experiments were carried out on Applied Biosystems ViiA7 Real-Time PCR System (ThermoFisher Scientific).

### Quantification of free amino acids through UPLC

For seed sample preparation, hard testa removed and five seeds enclosed in 1.5ml tube were ground to flour in a FastPrep homogenizer (Thermo Electron, FP120). One ml ethanol (80%, diluted in HCl) and 8 µl norvaline (2.5mM) were added to the cotton flour, incubated at 55 °C for 30 min and centrifuged at 18,000 × *g* for 10 min. The 100 µl supernatant was purified in Ultrafree-CL centrifugal filter units (Durapore-PVDF 0.22 µm) at 18,000 × *g* for 5 min, vacuum dried at 60 ^o^C for 1h and resuspended in 100 µl. For derivatization; 50 µl sample, 30 µl borate buffer, 20 µl AccQTag Ultra derivatizing reagent were mixed at room temperature for 1min, incubated at 55 ^o^C for 10 min and then analyzed.

Fresh phloem sap was collected from cotton petioles (five different leaves; 2cm) by putting into 0.2ml PCR tube to which a tiny hole was made and placed in another PCR tube without hole and the assembly was put together into a 1.5ml eppendorf tube. The liquid phloem sap was collected by centrifugation at 20,000 × g for 20 min, dried using Christ-RVC 2-18 CD-Plus vacuum concentrator at 60 ^o^C and resuspended in 20 µl borate buffer. The sample was derivatized by taking 10 µl sample, 60 µl borate buffer, 10 µl Norvaline and 20 µl AccQTag Ultra-derivatizing reagent, mixed at room temperature for 1min, incubated at 55 ^o^C for 10min and then analyzed at Waters Acquity UPLC, Tunable UV (TUV) Detector (Waters, Milford, USA).

### Morphological analysis of cotton plants and cotton fiber

The plant height, total number of bolls, sympodial/monopodial branches, and total fiber yield/plant were measured in T0 (April–Nov 2016, Lahore, Pakistan) and T1 generations (Feb 2017–Feb 2018, Denmark) of cotton plants (transgenic vs. non-transgenic). Fiber samples were sent to CCRI Multan for analysis of fiber length, micronaire value, fiber strength, and uniformity index. Whereas they were sent to the Centre for Electron Microscopy, Zhejiang University, China for SEM analysis.

### Physiological analysis

Net carbon assimilation/respiration (*A*), transpiration (*E*), and stomatal conductance (*g*_*s*_)were measured with an infrared gas analyzer CIRAS-3 of PP-systems in July–August 2018, when the atmospheric relative humidity ranged between 65–84%.

### *In planta* whitefly bioassay

Clip-cages were used for observing whitefly mortality in the T2 generation of cotton plants where the plants were raised for two months in special net cages to protect them from any insect infestation. Young cotton leaves were exposed to newly emerged whitefly adults (0–24 h) encased in clip-cages to prevent their escape. On the third day, any live whitefly was removed from the cage, the mortality data was recorded, and the area on the leaf was marked. The plants were quarantined in specialized net cages for observation of nymph development on tag leaves. Nymphs and adults of the next generation of whiteflies were followed up further to observe any effects of aspartic acid. Leaves were detached from plants and observed under the microscope to detect any hatched adults or nymph development. Moreover, the leaves subjected to the assay, were observed for the appearance of virus symptoms, which was expected to reflect the whitefly population indirectly.

### Fluorescence *in situ* hybridization

Fluorescence *in situ* hybridization (FISH) analysis was performed to determine the transgene location on the chromosome. The transgene was detected by labelling the probe with the Label IT Nucleic Acid Labeling kit (Mirus Bio LLC), Cy3, per manufacturers’ instructions. *In situ* hybridization was carried out by following protocols as described by^62^ on metaphase chromosomal spreads. Fluorescent signal detection was performed using a fluorescent microscope (Olympus Model BX6l). Blue (DAPI) and red^63^ filters were used to detect fluorescent signals.

## Supplementary information

Supplementary Information.
