# Supplementary material for: Constitutive expression of Asparaginase in Gossypium hirsutum triggers insecticidal activity against Bemisia tabaci
Source: Sci Rep. 2020 Jun 2;10:8958. doi: 10.1038/s41598-020-65249-w (PMC7265412; doi:10.1038/s41598-020-65249-w)
Supplement: Supplementary file 1 — Supplementary Information. [file 41598_2020_65249_MOESM1_ESM.docx]

**Constitutive expression of Asparaginase in *Gossypium hirsutum* triggers insecticidal activity against *Bemisia tabaci***

**Ambreen Gul^a^****^†^, Ghulam Hussain^a^, Adnan Iqbal^a^, Abdul Qayyum Rao^a†^*, Salah ud Din^a^, Aneela Yasmeen^a^, Naila Shahid^a^, Ammara Ahad^a^, Ayesha Latif^a^, Saira Azam^a^, Tahir Rehman Samiullah^a^, Samina Hassan^a,b^, Ahmad Ali Shahid^a^, Tayyab Husnain^a^.**

**Supplementary Data**

**Supplementary I**. **Agarose gel electrophoresis (1%) for screening of positive *ZmASN* clones by: (A)** Restriction digestion of pCAMBIA35S_*ZmASN* plasmid transformed in *E. coli* top10 with *Hind*III and *Sac*I restriction enzymes. Lanes 1–5 and 7–10: restriction digests; Lane 6: 1 kb DNA marker; 1562 bp represents the digested fragment size and is indicated by red arrow. **(B)** Gene specific PCR of pCAMBIA35S_*ZmASN* transformed into *Agrobacterium tumefaciens* LBA4404. Lane 1: Positive control; Lane 2: PCR negative control; Lane 3: 1Kb Marker; Lanes 4–6: selected colonies. Amplicon length is 736 bp and is indicated by red arrow.

**Supplementary II.** **PCR screening of putative transgenic Cotton plants in T0 generation**. PCR screening of *ZmASN* plants in T0 generation. Lanes 1, 10, 18, and 28: 1 kb marker; Lanes 8, 11, and 19: PCR negative control; Lanes 9, 17, and 20: positive control; Lanes 6, 16, and 21: Nontransgenic plant; Lanes 2–5, 7, 12–15, and 22–27: putative transgenic plants where 1564 bp represents the amplicon length. (NB: Lane 1-9, 10-17 & 18-28 represent three different screening gels imaged at different times, hence all controls and DNA markers are loaded separately in each gel).

**Supplementary III. Morphological characteristics of *ZmASN* transgenic plants. A-0, B-0, C-0 and D-0** represent data in T0-generation while **A-1, B-1, C-1** and **D-1** represent data in T1 generation. Control plant is the non-transgenic cotton plant. Data were statistically analyzed using one-way ANOVA and Dunnett’s multiple comparison test. The **** indicates significant data determined at *p*<0.0001. Data was recorded as *n* + SD whereas, *n* = 3.

**Supplementary IV. Leaf Area Index** of transgenic Cotton lines as compared to non-transgenic cotton lines. The leaves were harvested from upper (2), middle (2) and lower sections of each plant. The graphs are plotted as means of five biological replicates + SD.

**Supplementary V. Fiber characteristics of transgenic cotton lines of *ZmASN* gene in comparison to the control plant**. Whereas, control refers to the non-transgenic plant and number 14 to 28 refers to the transgenic lines. **A)** UHML or fiber length in mm. **B)** Fiber strength **C)** Micronair value of control and transgenic cotton lines **D)** Uniformity Index of control and transgenic cotton lines. Data were statistically analyzed using one-way ANOVA and Dunnett’s multiple comparison test. The **** indicates significant data determined at *p*<0.0001. Data was recorded as *n* + SD whereas, *n* = 3.

**Supplementary VI. Photosynthetic measurements in *ZmASN* transgenic lines.** (**A**) Net photosynthesis (*A*). (**B**) Stomatal Conductance (*g_s_*) and (**C**)Transpiration (*E*) of *ZmASN* Transgenic lines. Data were statistically analyzed using one-way ANOVA and Dunnett’s multiple comparison test. Data was recorded as *n* + SD whereas, *n* = 3. The **** indicate significant data determined at *p*<0.0001.

**Supplementary VII.** Whitefly biotype determination through PCR. The red arrow indicates the amplified fragment size of 303bp for Q biotype. Lane 1, 2 and 4: PCR with B-biotype primers; Lane 3, 5, 6: PCR with Q-Biotype primers; Lane 7: negative control; Lane 8: 50bp DNA marker.

**Supplementary VIII.** All primer sets used in this study.


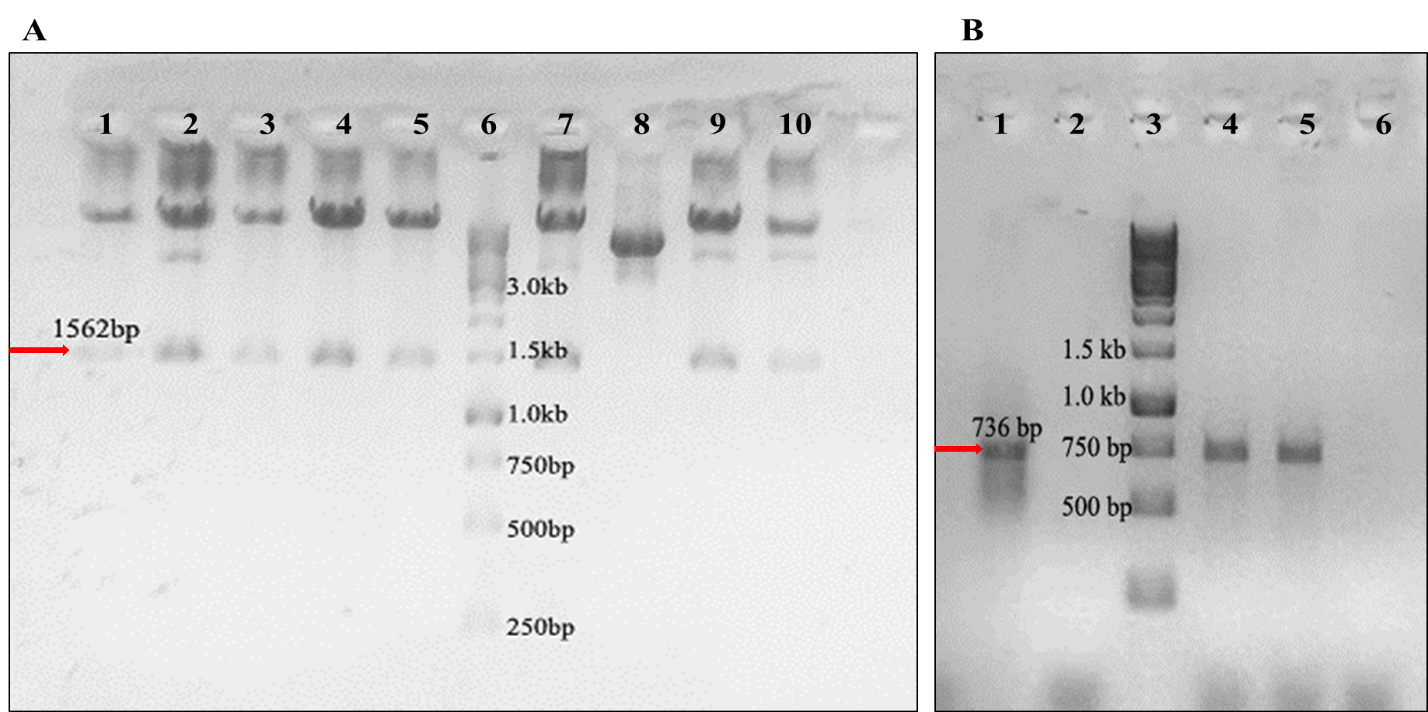


**Supplementary I**


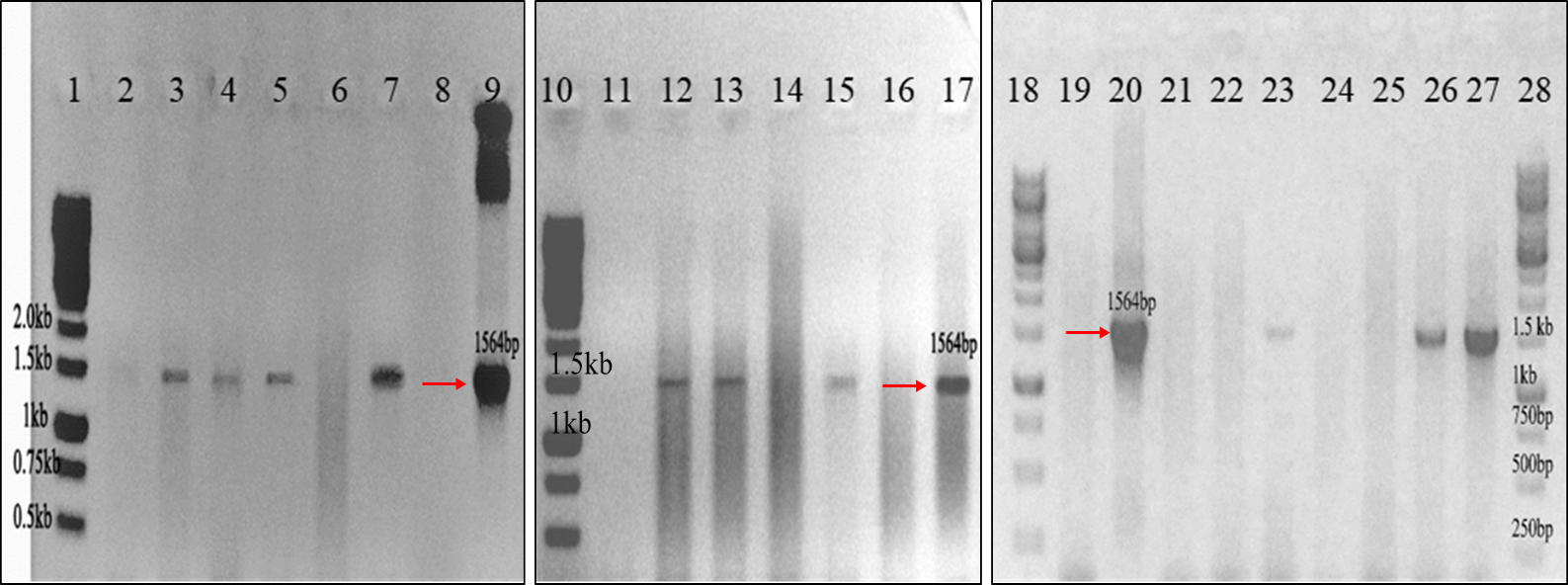


**Supplementary II**

**
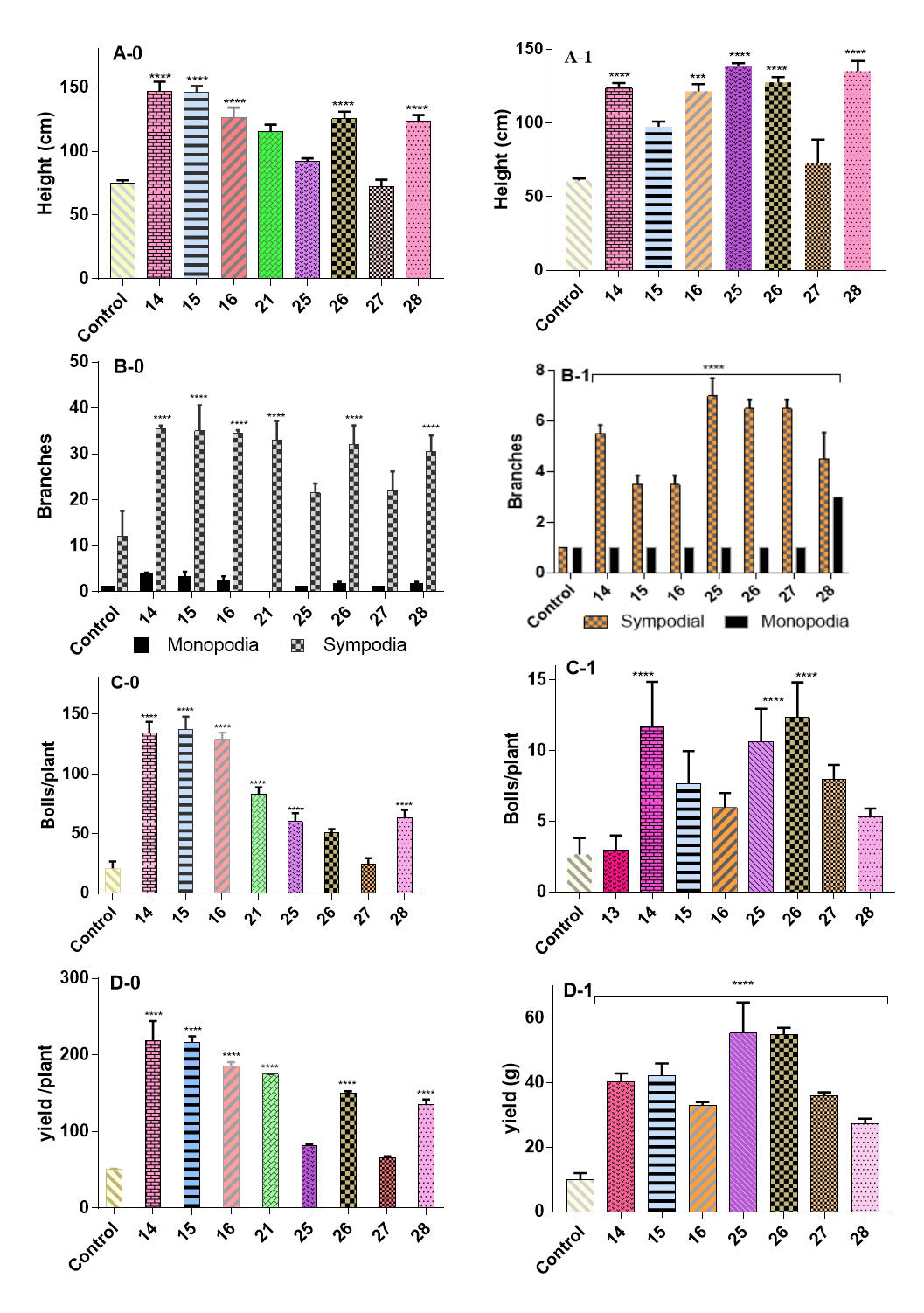
Supplementary III**

**Supplementary IV**

**Supplementary V**


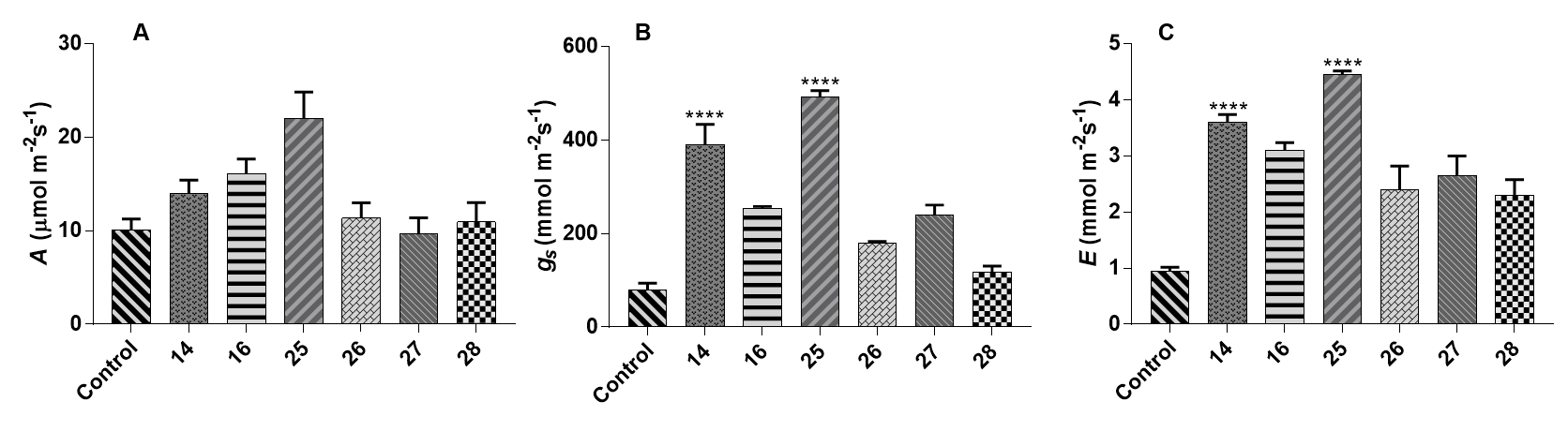


**Supplementary VI**

**
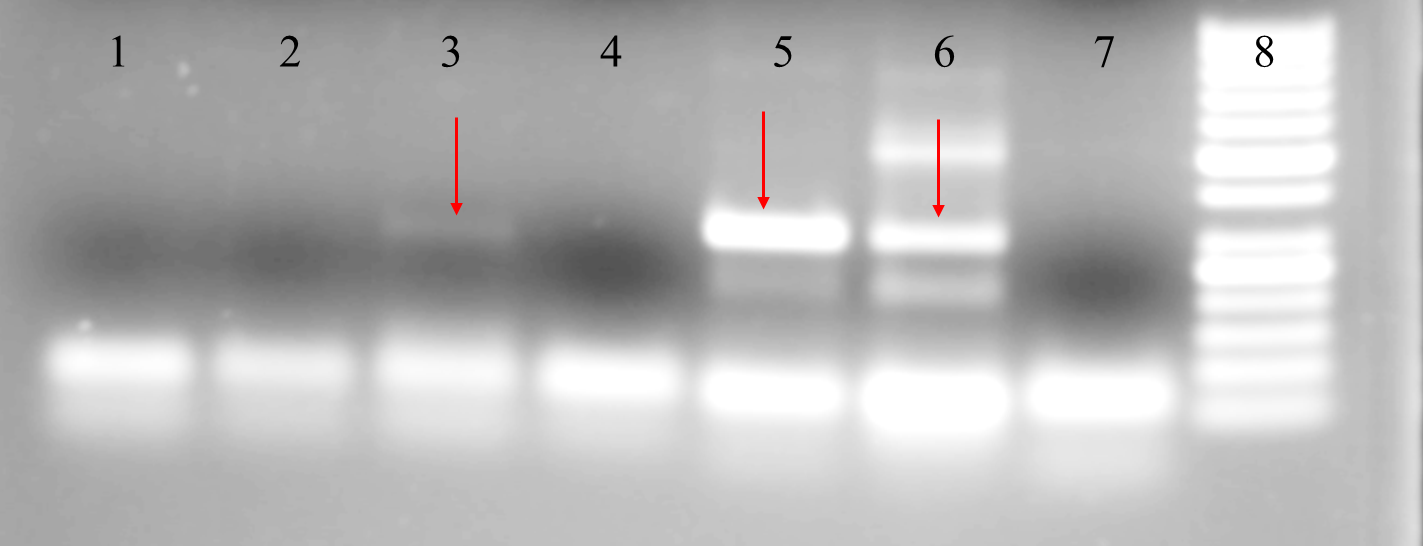
**

**Supplementary VII**

**Supplementary VIII**

| **S. No./name** | **Primer Sequence 5’ to 3’** | **Amplicon size (bp)** |
| --- | --- | --- |
| 1. *ZmASN*-full | GAGCTCTGAGACTTTTCAACAA | 1564 |
|  | AAGCTTTGATTCGTTACCGC |  |
| 1. *ZmASN*-short | TGGACGGTTCTACAGGTTC | 736 |
|  | TGATTCGTTACCGCTTATTGG |  |
| 1. *ZmASN*-qRT | CTTCTATAATGGACGGTTCTACAGG | 173 |
|  | AGCCCACTGTTAGGCACGTTC |  |
| 1. GhAct4 | TTGCAGACCGTATGAGCAAG | 105 |
|  | ATCCTCCGATCCAGACACTG |  |
| 1. Q-biotype | CTTGGTAACTCTTCTGTAGATGTGTGTT | 303 |
|  | CCTTCCCGCAGAAGAAATTTTGTTC |  |
| 1. B-biotype | CTAGGGTTTATTGTTTGAGGTCATCATATATTC | 478 |
|  | AATATCGACGAGGCATTCCCCCT |  |
